# Conference report: CHINAGUT 2023

**DOI:** 10.1002/imt2.153

**Published:** 2023-11-27

**Authors:** Danyi Li, CHINAGUT 2023 Organizing Committee

**Affiliations:** ^1^ R Institute Co., Ltd. Beijing China

## Abstract

CHINAGUT 2023, the premier conference on gut research in China, gathered over 10,000 participants in Beijing. Spanning 14 tracks and 35 sessions, it featured presentations from more than 300 scholars, over 400 presentations, and displayed 1000 posters, highlighting global advancements in the field. Photo by the R Institute.

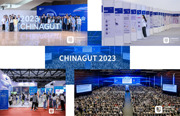

## A GLANCE OF CHINAGUT 2023

The CHINAGUT Conference, a biennial event dedicated to gut science, has grown significantly since its inception in 2018. Its 4th iteration, CHINAGUT 2023, was held from May 19 to 22 at the Beijing National Convention Center, attracting over 10,000 attendees. This large‐scale academic interchange, organized by numerous academic and industrial entities, has become China's premier conference on gut‐related research.

This year's conference showcased an evolved content structure with a comprehensive call for submissions, channeling over 60% of oral presentations from submitted abstracts. The conference was organized into 14 major academic tracks reflecting hot research fields, including Microbiome, Translational Medicine, Nutrition and Food, Animal Gut, Immunology, Metabolism, Neurology, Gut Physiology and Development, Oncology, Inflammatory Bowel Disease (IBD), Holistic Integrated Enterology, Special Populations, Technology and Methods, and One Health, with two additional augment tracks on Career Development and Public Science Education. The event hosted 35 academic sessions, drawing over 300 global scholars contributing to over 400 presentations and exhibiting about 1000 posters.

This conference report navigates through the highlights of the conference, chiefly focusing on plenary presentations, salient points from concurrent sessions, and the accolade of awards.

## PLENARY PRESENTATIONS

The opening ceremony highlighted seven keynote presentations, unveiling a rich tapestry of advancements in gut science research and industry. Covering a broad spectrum from gastrointestinal cancer, metabolic diseases, and intestinal immunity to the gut–lung axis, microbial interventions, cohort studies, and nutritional epidemiology, these talks collectively spotlight the interdisciplinary evolution of gut science, showcasing innovative pathways for solutions and therapies.

### Frontiers of microbiome and probiotics in cancer, Prof. Jun Yu, The Chinese University of Hong Kong

Prof. Jun Yu explored the link between gut microbiota and tumorigenesis, unveiling a distinct microbial signature in colorectal cancer (CRC) through meticulous multicohort analysis. She highlighted the identification of specific bacteria promoting CRC and others like *Streptococcus thermophilus* inhibiting CRC via anti‐CRC enzymes or metabolites production. Prof. Yu emphasized the potential of composite probiotics in CRC prevention and treatment and their role in enhancing anti‐PD‐1 immunotherapy efficacy against CRC. She also discussed microbiome alterations across different stages of gastric cancer, hinting at novel therapeutic strategies for gastrointestinal cancers and underscoring the significant clinical implications of microbiome research in cancer treatment [1].

### Gut microbiota and metabolic diseases, Prof. Changtao Jiang, Peking University

Prof. Changtao Jiang delved into the impact of gut microbiota on metabolic diseases and highlighted the significant mediation of microbial small molecule metabolites [2]. He explored detailed examples of bile acids, such as glycodeoxycholic acid, glycoursodeoxycholic acid, and hyocholic acid, and their distinct mechanisms in the development or prevention of various metabolic diseases like polycystic ovary syndrome, obesity, type 2 diabetes, and nonalcoholic fatty liver disease. He also revealed a microbial mechanism degrading nicotine, suggesting a new target to mitigate smoking‐related metabolic disorders [3]. These findings open avenues for potential therapeutic strategies in combating metabolic diseases by understanding the gut microbiota–host metabolism interplay.

### Impact of gut microbiome in respiratory tract infections and COVID‐19, Prof. Siew Ng, The Chinese University of Hong Kong

Prof. Siew Ng discussed the complex interplay between the gut microbiome and COVID‐19, emphasizing its role in host immunity and disease severity [4]. She introduced a specially designed Asian‐targeted microbiome immunity formula (SIM01) that showed promise in reducing adverse vaccine reactions and alleviating COVID‐19 symptoms like digestive issues in clinical studies. The formula's effectiveness is likely due to the regulation of gut microbial composition and its metabolites. Prof. Ng's insights spotlight the potential of gut microbiota modulation as a novel approach to COVID‐19 prevention, treatment, vaccine response enhancement, and addressing long COVID challenges.

### Organization and practice of cohort‐based microbiome research project, Prof. Jingyuan Fu, University of Groningen

Prof. Jingyuan Fu highlighted the value of large cohort studies in dissecting the gut microbiota's impact on health and diseases, using the Lifelines cohort of nearly 170,000 individuals as a case in point [5]. Despite challenges in experimental design and data management, these studies pave the way for personalized medicine by analyzing the interplay of genetics, diet, environment, and socioeconomic factors with gut microbiota on various diseases and traits. Moreover, Prof. Fu's team leverages induced stem cell and organ‐chip technologies to advance research in coculturing microbiota with human cells, promising new insights for managing health and diseases through a better understanding of the gut microbiome.

### Nutritional epidemiology: New findings, progresses and challenges, Prof. An Pan, Huazhong University of Science and Technology

Prof. An Pan shed light on the challenges and advancements in nutritional epidemiology [6]. He pinpointed issues like inaccurate dietary measurements and confounding factors. To surmount these hurdles, innovative solutions such as “Internet Plus” technology and Artificial Intelligence (AI) image recognition should be applied. He also discussed novel analysis models like gene–nutrition interactions, diet–microbiota interactions, and multiomics approaches. Novel intervention methods leveraging the Internet of Things and AI are highlighted as well. He stressed that the trajectory of nutritional epidemiology is steering toward a more individual‐centric approach, emphasizing personalized nutrition therapeutics as the future. His insights mark a significant stride toward a deeper understanding and improved application of nutritional epidemiology in public health.

### Spatial characteristics of intestinal immune system, Prof. Shu Zhu, University of Science and Technology of China

Prof. Shu Zhu explored the spatial characteristics of the intestinal immune system, emphasizing spatial variance in the types and differentiation of intestinal epithelial cells [7]. He highlighted the distinct roles of multiple gut regions, like, the epithelium, lamina propria lymphocytes, mesenteric lymph nodes, and Peyer's patches. Prof. Zhu discussed the interconnectedness between the intestinal immune system, enteric nervous system, and microbiota and their collective impact on health. He also elaborated on how immune cell composition, tissue properties, and environmental factors influence intestinal immunity's metabolic aspects. This exploration sheds light on the complex gut microbial, food, and immune crosstalk, hinting at potential new therapies for digestive and systemic diseases.

### Release of “2023 Gut Industry Development White Paper”, Liuyiqi Jiang, Zhejiang University

Liuyiqi Jiang introduced the “2023 Gut Industry Development White Paper,” revealing a 10‐sector gut industry driven by favorable policies, advanced technology, and health demands. Highlighting recent breakthroughs extending from fecal microbiota transplantation (FMT) to microbial therapeutics for various diseases, the presentation underscored China's leadership in probiotic research and the approval of numerous special medical foods. Additionally, the industry's focus extends beyond human health, addressing ecological agriculture and pet health challenges and showcasing a holistic approach toward health and nutrition.

## HIGHLIGHTS OF CONCURRENT SESSIONS

### Submission overview

The CHINAGUT 2023 conference showcased a significant volume of cutting‐edge research through a rigorous call for papers, which were meticulously reviewed by experts to select the crème de la crème for oral presentations at the event. The call drew 854 abstract submissions, with nearly 600 of them being unpublished studies. The geographical spread of the submissions, coming from 22 countries, including the United States, the Netherlands, Canada, the United Kingdom, Germany, and Sweden, among others, signifies the growing international influence of CHINAGUT.

**Figure 1:**
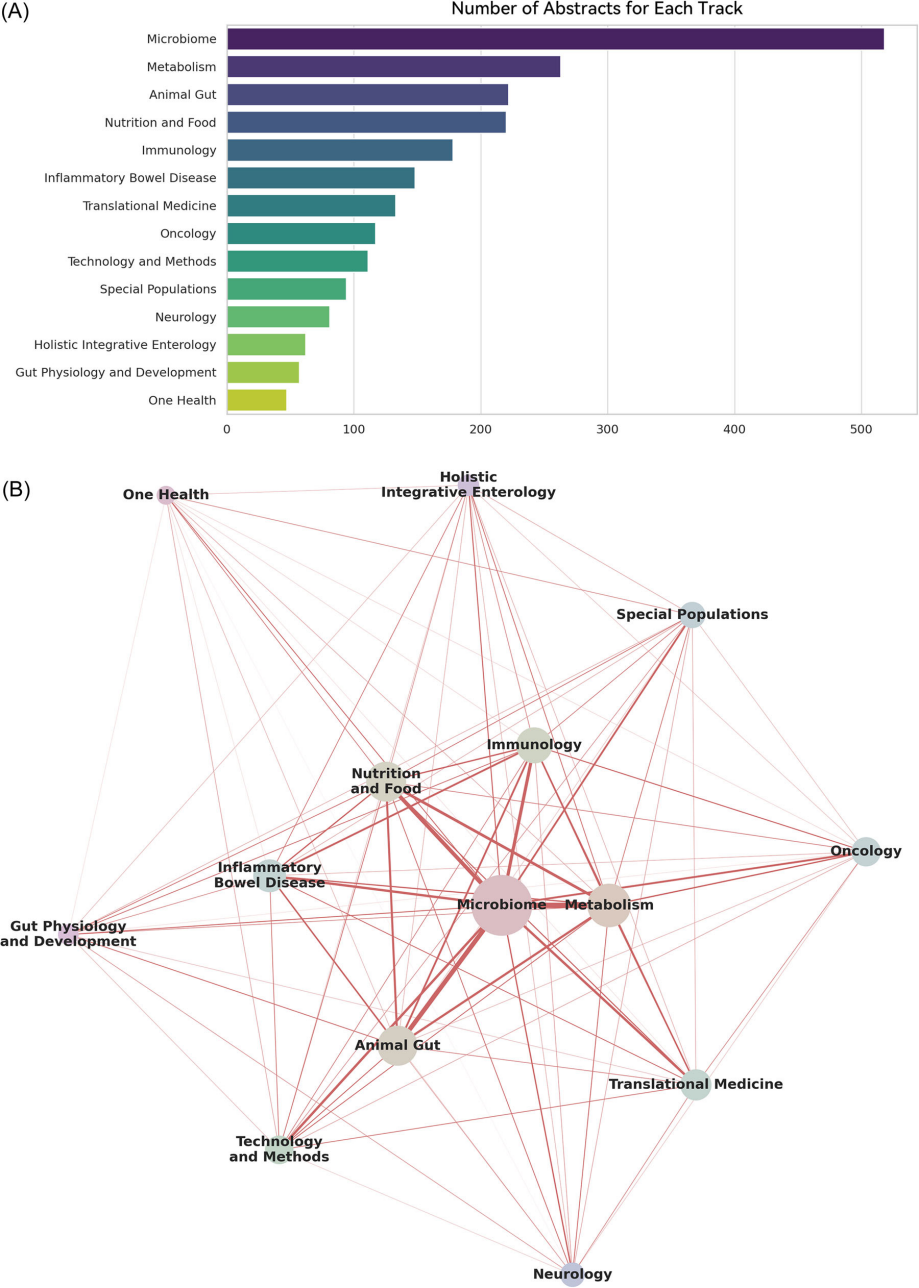
Overview of abstract submission. (A) Numbers of abstracts submitted for each track. (B) Cooccurrence network of the 14 tracks. The size of the nodes represents the number of abstracts for each track, while the edges (connecting lines) indicate the cooccurrence of two tracks. The thickness of the edges reflects the number of cooccurrences.

The analysis of the submissions in each track painted a sketch of the current hotspots in gut science research, particularly shedding light on the focal points within China's research community (Figure 1A). Microbiome emerged as the predominant theme, reflecting the growing interest in understanding the complex microbial communities residing in the gut and their profound impact on host health. Traditional foundational research areas such as Metabolism and Immunology continue to be pivotal, providing essential insights into the gut's role in broader physiological contexts.

Furthermore, themes like Animal Gut, exploring model animals, economic animals, wild animals, and pets, are gaining traction, underlining the importance of a One Health approach. Notably, tracks like Nutrition and Food and Translational Medicine are brimming with transformative research poised to bridge the bench‐to‐bedside gap. IBD remained a focal point of disease‐oriented research, indicative of the persistent quest for understanding and alleviating gut‐related ailments.

Moreover, a network analysis of multitrack cooccurrences illustrated the interdisciplinary synergy among the current research endeavors (Figure 1B). For instance, a close interlink was observed among Microbiome, Metabolism, Animal Gut, and Nutrition and Food tracks, reflecting a high degree of relevance and collaboration among these domains. This intertwining of themes not only underscores the multifaceted nature of gut science research but also highlights the burgeoning focal points and collaborations driving the current wave of investigations in this sphere.

### Overview and highlights of concurrent sessions

#### Inflammatory bowel disease

May 19, four sessions

Track: Inflammatory Bowel Disease

Chaired by Prof. Zhanju Liu and Prof. Yulan Liu

The Inflammatory Bowel Disease (IBD) sessions featured 14 insightful reports focusing on the pathophysiological mechanisms of IBD. The core of discussions revolved around the roles and molecular mechanisms of gut microbiota, host genes and proteins, immune cells, and bile acids in IBD, aiming to unveil new therapeutic targets and strategies. Key topics included host–microbe interactions, microbial biomarkers in IBD, and the fusion of AI with precision medicine in IBD research. The keynotes enriched the session with Prof. Zhanju Liu exploring the interplay between host genes and bacteria in IBD onset and precise diagnosis, Prof. Ruixin Zhu delving into the advancements and challenges in IBD microbial biomarkers, Prof. Zhen He discussing targeting mesenteric microbiota for Crohn's Disease, Prof. Bangmao Wang examining the interaction between bile acids and colitis and cancer, and Prof. Weiguo Dong shedding light on the role of Fusobacterium nucleatum in Ulcerative Colitis.

#### Technology and methods

May 20, two sessions

Track: Technology and Methods

Chaired by Prof. Xiaodong Su and Prof. Jian Xu

The Technology and Methods sessions witnessed 12 presentations (including six mini‐talks). Microbial single‐cell technologies took center stage with discussions on high‐throughput microbial genomics, microfluidic single‐cell techniques for precise microbial culturing, and FlowRACS for rapid profiling of the metabolic function of microbiota at single‐cell resolution. Imaging technologies unveiled advancements in spatial microbiome imaging and in vivo fluorescence probe‐based imaging for microbiome research and microscopic imaging for colitis‐cancer progression. AI was highlighted for microbial gene function prediction and AI‐enhanced colonoscopy. Application of Raman spectroscopy, in vitro human gastrointestinal system, and antimicrobial peptide design showcased novel strategies were also touched, marking the session a hotbed of innovative methodologies propelling gut science.

#### Aging and gut health

May 20, two sessions

Track: Special Population

Chaired by Prof. Qiang Zeng and Prof. Chenhong Zhang

The Aging and Gut Health sessions featured 10 presentations (including three mini‐talks), delving into the intersection of aging and gut science. Prof. Yangfeng Wu highlighted low‐sodium salt as a key strategy against hypertension and cardiovascular diseases, grounded in clinical and real‐world evidence. Prof. Lemin Zheng elucidated the connection between gut microbiota metabolism and cardiac aging, emphasizing the impact of the microbiota‐derived metabolite N,N,N‐trimethyl‐5‐aminovaleric acid on cardiometabolic diseases tied to aging. Other talks explored the ties between gut microbiota and aging‐related chronic conditions, and the potential of nutritional and microbiota interventions for anti‐aging and disease prevention, illuminating the synergy between dietary approaches, microbial communities, and aging wellness.

#### One health

May 20, two sessions

Track: One Health

Chaired by Prof. Gong Cheng and Prof. Ruifu Yang

The One Health sessions, comprising nine presentations, were convened to spotlight the interconnection of human, animal, and environmental health. Prof. Jianguo Xu, in his keynote, unraveled the complexity of the normal human microbiota using metataxonomics, highlighting the prevalence of pathobionts in the healthy human gut and the gut microbiota distinctions between humans and various wild animals. The session encapsulated critical discussions on zoonotic diseases, vector‐borne diseases, pathogen dynamics, antibiotic resistance, and extracellular vesicles, underscoring the quintessence of the “One Health” paradigm in health research. Moreover, it showcased the application of microbiomics in deciphering infectious disease transmission and antimicrobial resistance issues, thereby fostering a holistic, interdisciplinary approach toward health and disease realms.

#### Metagenomics

May 20, two sessions

Tracks: Technology and Methods, and Microbiome

Chaired by Prof. Yong‐Xin Liu and Prof. Jun Wang

The Metagenomics sessions, featuring 10 presentations, delved into the expansive field of metagenomics with a particular emphasis on quantitative analysis to unravel human gut microbiota ecology and discern microbiome–disease–drug interactions. Notable attention was given to strain‐level microbe tracking, shedding light on microbial transmission within and between individuals. Significant advancements in software and algorithm development were showcased, aiding in precise metagenomic analyses and microbial metabolic background matching. The exploration of causal relationships between gut microbiota and blood metabolites, as well as between microbiota and human diseases, stood out, hinting at potential therapeutic avenues. Moreover, the sessions touched on gut virome analysis, offering a glimpse into viral diversities within the gut ecosystem. Through these focal points, these sessions underscored the advancement of metagenomics in dissecting the intricate interplay between microbiome and host health.

#### Gut–brain axis

May 20, two sessions

Track: Neurology

Chaired by Prof. Xingyin Liu, Prof. Hongwei Zhou, and Prof. Fangqing Zhao

In the Gut–Brain Axis sessions, a total of 10 riveting presentations were delivered, honing in on the intricate interplay between microbiota and the gut concerning neurological studies and related disorders. The spotlight was on an array of mental and neurological conditions, like, depression, autism spectrum disorders, and Alzheimer's disease. This session underscored the multifaceted explorations in the microbiota–gut–brain axis domain, unveiling the regulatory role and mechanisms of microbiota on brain health, such as metabolites, inflammation, and RNA editing. Moreover, it highlighted the translational potential of drug development, probiotics, dietary interventions like intermittent fasting, and the amalgamation of traditional Chinese and Western medicine in tackling mental and neurological ailments, thereby paving the way for innovative therapeutic strategies.

#### Holistic integrated enterology

May 20−21, four sessions

Track: Holistic Integrated Enterology

Chaired by Prof. Kaichun Wu and Prof. Faming Zhang

With 24 presentations (including 10 mini‐talks), the Holistic Integrative Enterology session spotlighted a spectrum of research avenues focused on basic, clinical, and translational studies addressing various gastrointestinal disorders and the interplay between the gut and other organs (gut–X axis) in health and disease, with a notable emphasis on gut microbiota. IBD and irritable bowel syndrome were the primary diseases discussed. Prof. Michael Kamm and Prof. Bo Shen as keynote speakers delved into advancement in IBD treatment. The sessions showcased the potential of microbiota transplantation and other clinical techniques in treating bowel diseases. It also ventured into the realm of drug and nutritional interventions, including Chinese medicine and probiotics, for disease management. Multifaceted omics analyses were employed to unravel the mechanisms underlying gut‐related disorders, offering a window into the complex gut–X axis interactions. The studies presented in this session underscored the pivotal role of multidisciplinary efforts in advancing the understanding and treatment of digestive system diseases.

#### Gut and traditional Chinese medicine (TCM)

May 21, two sessions

Track: Translational Medicine

Chaired by Prof. Hongwei Liu, Prof. Zhaoxiang Bian, and Prof. Yan Wang

Gut and TCM sessions, consisting of 15 presentations (including five mini‐talks), delved into the intersection of gut health and TCM. The keynote address by Prof. Xudong Tang focused on the evaluation and elucidation of treatment mechanisms for lactose intolerance from the perspective of the spleen deficiency theory. A significant part of the discussions revolved around the interactions between gut microbiota and TCM compounds, particularly in the treatment of digestive disorders. Emphasis was also placed on the novel pharmacological actions and mechanisms of TCM in disease treatment, with a keen interest in polysaccharides from Chinese medicine. These explorations contribute to a deeper understanding of how TCM, through modulating gut microbiota, can offer therapeutic benefits, thus providing insights into new treatment strategies and drug discoveries.

#### Gut physiology and development

May 21, two sessions

Track: Gut Physiology and Development

Chaired by Prof. Zhihua Liu and Prof. Zhengquan Yu

Comprising 12 presentations, including five mini‐talks, these sessions delved into crucial realms of gastrointestinal research, underpinned by three core themes: gut immune regulation, microbial–gut interactions, and intestinal cellular biology. In their keynote talks, Prof. Zusen Fan discussed the discovery of new immune cell subgroups and their role in gut immune regulation, while Prof. Xiaoyu Hu elucidated the regulatory functions of intestinal macrophages in maintaining homeostasis. Additionally, microbial interplay emerged as a pivotal focus, exploring how gut microbial signals and metabolites influence physiological and pathological processes and regulate gut barrier function. The cellular aspect delved into the biology and functionality of various gut epithelial cells and the molecular signaling pathways governing intestinal inflammation, cell differentiation, and tissue repair. These multifaceted explorations deepened the understanding of gut physiology and pathophysiology, fostering a solid groundwork for potential therapeutic advancements in gastrointestinal disorders.

#### Evidence‐based nutrition

May 21, four sessions

Track: Nutrition and Food

Chaired by Prof. Hanping Shi, Prof. An Pan, and Prof. Jusheng Zheng

Incorporating 16 presentations, the sessions on Evidence‐Based Nutrition meticulously examined the intertwined relations among dietary patterns, nutrition, gut microbiota, and various chronic diseases, such as cardiovascular diseases, cancers, and depression. The focal points included nutritional epidemiology, interactions between diet and commensal microbiota, dietary interventions for disease prevention and treatment, and the significance of precision and digital nutrition research in modern health management. Three keynote speeches spearheaded the discussion: Prof. Gangqiang Ding highlighted the health benefits of prebiotics and their impact on the food industry, Prof. Ying Li focused on digitizing basic nutritional studies within the framework of precision nutrition, and Prof. Dongliang Wang underscored the role of gut microbial metabolites in cardiovascular diseases. The sessions further discussed the repercussions of dietary patterns on environmental and public health, presenting a holistic exploration of dietary strategies tailored for both individual and public health improvement.

#### iMeta author forum

May 21, two sessions

Track: Technology and Methods

Chaired by Prof. Yong‐Xin Liu and Prof. Tong Chen

Most of the nine presentations at the iMeta Author Forum showcased eye‐catching publications from the iMeta journal [8]. This forum explored various aspects of microbiome analysis, bioinformatics visualization, and the development of analytical tools [9]. A central focus was on examining microbial interactions with hosts, including both plants and animals. Discussions also ventured into quantitative and low‐biomass microbiome analysis, coupled with the innovative use of bioinformatics, such as generating complex heat maps. These discussions were driven by a strong interest in utilizing modern bioinformatics tools to foster a deeper understanding and more effective dissemination of scientific data [10].

#### Gut microbiome

May 21, four sessions

Track: Microbiome

Chaired by Prof. Shuangjiang Liu and Prof. Changtao Jiang

Comprising 21 presentations, including three mini‐talks, the Gut Microbiome sessions explored the multifaceted and layered frontier topics of gut microbiome research. The keynote speeches by Prof. Chun‐Jun Guo and Prof. Harris Wang elucidated the molecular mechanisms of microbiota–host interactions using gut microbial gene‐editing tools and highlighted the automation in microbiome culturomics based on machine learning, respectively. A notable focus was on the roles and underlying mechanisms of gut microbiota in various diseases and conditions, such as autoimmune liver disease, diabetic peripheral neuropathy, weight rebound, alcoholic hepatitis, cancer, and rheumatoid arthritis. The discussions further extended to the exploration of microbial metabolites and natural products, along with their biosynthesis mechanisms. Research resources and methods like ultra‐deep sequencing and multiomics approaches were also highlighted. The sessions also illuminated microbial dark matter, encompassing fungi and phages. The engaging discourse, grounded in a multidisciplinary approach, epitomized the profound scope of gut microbiome research, unveiling new vistas for therapeutic strategies and advancements in microbiome science.

#### Gastrointestinal tumor

May 21, two sessions

Track: Oncology

Chaired by Prof. Jun Yu, Prof. Jingyuan Fang, and Prof. Sunny Wong

The sessions, encompassing 12 presentations including three mini‐talks, delved into a comprehensive and multidimensional exploration of gastrointestinal cancer‐related research. This exploration touched on a variety of cancers, including but not limited to colorectal, hepatic, and esophageal cancers, spanning microbiological, immunological, metabolic, and nutritional perspectives to understand cancer genesis, evolution, and therapeutic avenues. The keynote speeches by Prof. Jingyuan Fang and Prof. Sunny Wong highlighted the intricate role of gut microbiota in modulating metabolism, thus impacting the clinical diagnosis and treatment of CRC, and the tripartite link between obesity, gut microbiota, and cancer progression, respectively. The discourse extended to the mechanisms through which microbiota and their metabolites engage in metabolic and immunological regulation, contributing to cancer onset and progression. Topics such as early‐onset CRC, temporal changes in microbiota characteristics in cancer, and innovative microbial interventions like next‐generation probiotics for ameliorating cancer treatment were among the myriad topics covered.

#### Maternal and child microbiome

May 21, two sessions

Tracks: Special Populations, and Microbiome

Chaired by Prof. Fangqing Zhao and Prof. Qinping Liao

The session featured 12 presentations, exploring three major topics. The first one was the profound implications of gut and vaginal microbiota on maternal and women's health during pregnancy. It tackled conditions such as sepsis, intrahepatic cholestasis, Polycystic Ovary Syndrome, pre‐eclampsia, and female infertility. Another major focus was the pivotal role of early‐life gut microbiota in infant ailments like neonatal jaundice and in longer‐term child health issues, including neurodevelopment, autism, and obesity. Moreover, the session delved into the potential interventions and mechanisms of specific microbes like *Akkermansia muciniphila* and *Bifidobacterium*, as well as their products like extracellular vesicles, shedding light on their impact on maternal and child health. Through a broad lens, these discussions unraveled the microbial interactions and their enduring effects on the health trajectory from pregnancy to childhood.

#### Soil and environmental microbiome

May 21, two sessions

Tracks: One Health, and Microbiome

Chaired by Prof. Haiyan Chu and Prof. Yanpo Yao

Soil and Environmental Microbiome sessions, comprising 11 reports, delved into the nexus of plant, soil, and environmental microbiomes. It spanned a variety of research themes, from microbial community structures and ecological functions to the impacts of human activities like fertilization and antibiotic use and ecological restoration. The discussions spotlighted the roles and applications of microbes in agricultural production, unveiling a new vista for sustainable agriculture through microbial insights.

#### Microbiota transplantation

May 21, two sessions

Tracks: Translational Medicine, and Microbiome

Chaired by Prof. Faming Zhang and Prof. Yongzhan Nie

The Microbiota Transplantation sessions featured 10 presentations, inclusive of four mini‐talks. Prof. Gianluca Ianiro delivered the keynote, exploring the dynamics of the microbiome following FMT across various diseases. A notable discussion centered around the application and efficacy of washed microbiota transplantation (WMT) in addressing a range of diseases, from refractory intestinal infections to metabolic disorders. The utility of FMT in the treatment of other ailments such as gastrointestinal motility disorder and hypertension was also highlighted. Moreover, the sessions delved into the pivotal factors concerning donors and recipients in microbiota transplantation. The potential of employing FMT as a robust research tool to unravel the profound influence of gut microbiota on host health and traits was also a significant focus of the discourse.

#### Gut immunity

May 22, two sessions

Track: Immunology

Chaired by Prof. Chen Dong, Prof. Noah Palm, and Prof. Shu Zhu

The Gut Immunity sessions provided a comprehensive insight into the complexities of the gut's immune responses and interactions with microbiota. Keynotes delivered by Prof. Kenya Honda, Prof. Chen Dong, and Prof. Noah Palm elaborated on interspecies communication for homeostatic microbiota structuring, the developmental plasticity of intestinal innate lymphoid cells, and mapping the uncharted domains of host–microbiota communication, respectively. Other topics predominantly focused on mucosal immunology encompassing microbiota–host interactions, covering aspects like food allergy, early life microbial exposure and B cell immunity, mucus sialylation in host‐commensal homeostasis, and gut pathobiont‐induced immune responses in diseases. The sessions also underscored the complex interplay among the gut microbiota, immune, and nervous systems. These discussions significantly contributed to the broader understanding of gut immunity and its interwoven relationship with microbial communities, paving the way for potential therapeutic interventions in gut‐related disorders.

#### Food innovation

May 22, two sessions

Track: Nutrition and Food

Chaired by Prof. Fengjiao Xin and Prof. Hao Zheng

The Food Innovation sessions, encompassing eight talks, delved into the nexus between food innovation, gut microbiota, and their implications for health and disease. A key highlight was the exploration of food processing, specific food components like dietary fibers and flavonoids, alongside microbes such as natural gut bacteria and engineered strains, in promoting digestive health and disease prevention. For instance, certain gut bacteria were spotlighted for their ability to mine active compounds hidden in fibers, thus unleashing the health benefits of the corresponding food. Additionally, advancements in rapid food safety detection and the design of bionic in vitro digestion absorption fermentation systems were discussed. The sessions also navigated the translational potential of nutritional interventions based on gut microbiota, adopting a food‐as‐medicine approach. These discussions are pioneering pathways for food innovation and delivering new scientific insights for enhancing digestive health.

#### Animal gut

May 22, four sessions

Track: Animal Gut

Chaired by Prof. Hong Wei, Prof. Haihong Hao, and Prof. Jiangchao Zhao

The sessions unveiled 18 insightful presentations, predominantly focusing on agricultural and wild animals. Discussions on agricultural animals elucidated the crucial interplay between microbiome, gut health, and animal nutrition, spotlighting microbiome‐targeting interventions. The keynote by Prof. Junjun Wang explored the nutritional modulation and the role of macrophages in intestinal barrier damage in low‐birth‐weight piglets. Alongside other talks, these discussions broadened the scope of agricultural animal research, encompassing swine, cows, poultry, and fish. Regarding wild animals, Prof. Mang Shi's keynote introduced metatranscriptomics for studying wild animal pathogens, offering fresh perspectives within the One Health paradigm. The gut microbiota of pandas also captured attention. Additionally, the session unveiled unique insights into the applications of germ‐free animals and intestinal organoids in animal research and advancements in isolating and culturing animal‐associated microbes. Through a blend of empirical findings and innovative methodologies, the session enriched the discourse around the diverse gut microbiome across the animal kingdom.

#### Gut and metabolism

May 22, two sessions

Track: Metabolism

Chaired by Prof. Liping Zhao, Prof. Wei Jia, and Prof. Leming Zheng

The Gut and Metabolism sessions, comprising eight presentations and two mini‐talks, spotlighted the intricate relationships between gut microbiota and various metabolic diseases, with a specific focus on diabetes. The discourse encompassed how gut microbial communities influence the onset and progression of metabolic disorders. A significant part of the discussion centered around the impact of microbial metabolites on host health, exemplified by the study on bacterial metabolite uridine diphosphate‐galactose enhancing pancreatic β‐cell function via the gut‐islet axis. The sessions also delved into drug and nutritional interventions for metabolic ailments, showcasing the application of Disulfiram and Liraglutide in ameliorating conditions like nonalcoholic fatty liver disease and type 2 diabetes, respectively. Additionally, innovative detection technologies like micro‐in‐one unveiled new perspectives in studying microcirculatory dysfunction in diabetic mice. Through these lenses, the sessions provided a rich narrative on the potential therapeutic strategies targeting the gut and the commensals within to mitigate metabolic diseases.

#### Microbial ecology symposium

May 22, two sessions

Track: Microbiome

Chaired by Prof. Lei Dai

The symposium unveiled a breadth of microbial ecology through nine insightful presentations, exploring intricate microbial interactions within communities. In his keynote speech, Prof. Yangyu Liu presented a Data‐driven Keystone species Identification framework, empowered by deep learning, to systematically pinpoint keystone species in microbial communities. Additionally, discussions extended to horizontal gene transfer, species coexistence, quorum sensing, synthetic communities, emergent properties of the microbial ecosystem, and cross‐kingdom interactions. The omics approach and big data analysis were prominently featured, providing insights into the microbe–host health nexus. This convergence of ideas fostered a rich understanding and unveiled novel avenues for delving into microbial ecology and translating its findings into practical applications.

#### Synthetic biology

May 22, two sessions

Track: Technology and Methods

Chaired by Prof. Chenli Liu and Prof. Lei Dai

The session, entailing seven presentations, primarily focused on the application of synthetic biology in engineering microbes. Engineered microbes such as engineered probiotics and phages, serving as live biotherapeutics for disease diagnosis and intervention, were the highlight of discussions. Prof. Matthew Chang, in his keynote talk, elaborated on their work on engineering gut‐resident bacteria to modulate host–microbiome interactions for therapeutic purposes. Furthermore, the frontier topic of engineering microbial consortia was also given notable attention. These presentations underscored the broad applications and innovative possibilities through synthetic biology techniques in microbial engineering, therapeutic development, and biopharmaceuticals, showcasing the vitality of synthetic biology in advancing microbiome‐centered research and applications.

#### Probiotics and prebiotics

May 22, two sessions

Tracks: Translational Medicine, and Nutrition and Food

Chaired by Prof. Wei Chen and Prof. Heping Zhang

The sessions featured 14 talks, including seven mini‐talks, exploring the expanding horizons of probiotics and prebiotics. Prof. Wei Chen's keynote delved into new advancements in probiotic research and applications from functional expansion, mechanistic insights, to broader application scopes and future trends. Prof. Heping Zhang, in his keynote, shared their team's basic and translational research experiences with two probiotic strains isolated from breast milk. The session revealed an extended application of probiotics and prebiotics beyond gut health improvement (e.g., constipation) to oral health, adjunctive treatment for CRC, and metabolic disease interventions (e.g., obesity and fatty liver). Emerging next‐generation probiotics, like, *A. muciniphila* and *Clostridium butyricum*, alongside traditional ones, were discussed. On the prebiotic front, besides dietary fibers, phenolic natural products emerged as a new research focus. Other highlights included quality control of probiotic products and probiotics in herbal medicine fermentation, offering fresh perspectives on the roles and applications of probiotics and prebiotics in promoting human health.

### Career development and more

Beyond the vibrant exchange of research insights, CHINAGUT 2023 curated an enriching avenue for professional development for its attendees. A series of meticulously structured training workshops were offered, delving into the realms of metagenomics, microbiome research quality control, and microbial single‐cell multiomics. A standout segment of the event was the Women Scientists Forum, where Prof. Jun Yu and Prof. Jingyuan Fu imparted their scholarly insights. They eloquently shared their journey, shedding light on the art of balancing the demanding roles of a scientist with the familial responsibilities inherent to being women. Their narrative resonated with many, offering a blend of inspiration and practical advice. The Young Scientists Forum served as a vibrant platform for young Principal Investigators to exchange experiences and establish collaborations. Moreover, in a collaborative endeavor with Wiley Publishing, a roundtable discussion was convened. Prof. Monty Montano, editor of the journals *Aging Cell* and *Advanced Biology*, alongside invited scientists from the realms of aging and TCM, shared their experiences, building bridges between researchers and academic journals.

## AWARDS

CHINAGUT has once again shone the spotlight on remarkable individuals and burgeoning talents in the field of gut health research and industry. The accolades were bestowed in three major categories, highlighting the remarkable work of both seasoned and emerging researchers and industry professionals.

The much‐anticipated R Awards, organized by the R Institute, were segmented into two categories: “Scientist of the Year” and “Gut Industry Person of the Year.” This year's distinguished “Scientist of the Year” award was shared by Prof. Changtao Jiang from Peking University for his groundbreaking work on gut microbiota and metabolic diseases, Prof. Faming Zhang from Nanjing Medical University for his clinical research in microbiota transplantation, particularly the development of WMT technique, and Prof. Wei Chen from Jiangnan University for his enduring commitment over two decades toward developing probiotics native to China, establishing the nation's largest proprietary probiotic strain bank. The “Gut Industry Person of the Year” was awarded to industry trailblazers—Mr. Guoxun Xiao, the founder and chief executive officer (CEO) of Wonderlab, Ms. Xiaoxu Sun, the Deputy General Manager of Mengniu Dairy Low Temperature Business Division, and Dr. Yan Tan, the CEO of Xbiome.

In its endeavor to nurture the next wave of researchers, R Scholarships were granted to 57 Chinese graduate students across various disciplines, including basic research, clinical medicine, and agrofishery and food science. A few of the honored scholars were Guangyi Zeng (Peking University), Qi Su (The Chinese University of Hong Kong), and Yunong Zeng (Guangdong Pharmaceutical University), among others.

Additionally, the Young Trainee Awards recognized 20 outstanding young researchers (graduate students and postdoctoral fellows) from a pool of the 854 submitted abstracts, each receiving a cash prize of CNY 2000. Some of the awardees include Dr. Daoming Wang (postdoctoral fellow, University of Groningen), Shujun Xu (graduate student, Hong Kong Baptist University), and Menglei Shuai (graduate student, Westlake University).

These awards not only underline the tireless efforts and novel findings of the awardees but also epitomize the vibrant and progressive landscape of gut health research and industry in China, setting a promising trajectory for the field.

## CONCLUDING REMARKS AND INVITATION TO CHINAGUT 2025

The CHINAGUT 2023 conference emerged as a fertile ground for the exchange of cutting‐edge research, innovative applications, and collaborative ventures in the realm of gut science and its associated industries, delving into various aspects of gut health‐related research and application, with a special focus on gut microbiome. The diverse sessions and forums catalyzed insightful discussions, while the professional development activities and the awards ceremony highlighted the burgeoning talent and remarkable contributions propelling the field forward.

On behalf of the CHINAGUT Organizing Committee, we cordially extend an invitation to the gut science community and the related industries to join us in the next chapter of this exploratory journey—CHINAGUT 2025. Set to be held from May 23 to 25, 2025, in China, the upcoming conference promises to further the discourse, foster lasting partnerships, and catalyze groundbreaking innovations that will continue to shape the landscape of gut science and its industrial applications.

## AUTHOR CONTRIBUTIONS

Danyi Li wrote and revised the manuscript. All authors have read the final manuscript and approved it for publication.

## CONFLICT OF INTEREST STATEMENT

The authors have declared no competing interests.

## Data Availability

The data that support the findings of this study are available from the corresponding author upon reasonable request. Supplementary materials (graphical abstract, slides, videos, Chinese translated version and update materials) may be found in the online DOI or iMeta Science http://www.imeta.science/.

## CHINAGUT 2023 Organizing Committee

(Alphabetize by Last Name)

Prof. Rui Bao (West China Hospital)

Prof. Zhaoxiang Bian (Hong Kong Baptist University)

Prof. Tong Chen (China Academy of Chinese Medical Sciences)

Prof. Wei Chen (Jiangnan University)

Prof. Xiaodong Chen (Soochow University)

Prof. Gong Cheng (Tsinghua University)

Prof. Haiyan Chu (Institute of Soil Science, Chinese Academy of Sciences)

Prof. Lei Dai (Shenzhen Institutes of Advanced Technology, Chinese Academy of Sciences)

Prof. Chen Dong (Shanghai Jiao Tong University)

Prof. Jingyuan Fang (Renji Hospital, School of Medicine, Shanghai Jiao Tong University)

Prof. Zhongze Fang (Tianjin Medical University)

Prof. Haihong Hao (Huazhong Agricultural University)

Prof. Wei Jia (Hong Kong Baptist University)

Prof. Changtao Jiang (Peking University)

Mr. Canhui Lan (R Institute)

Prof. Qinping Liao (Beijing Tsinghua Changgung Hospital)

Prof. Chenli Liu (Shenzhen Institutes of Advanced Technology, Chinese Academy of Sciences)

Prof. Hongwei Liu (Institute of Microbiology, Chinese Academy of Sciences)

Prof. Shuangjiang Liu (Institute of Microbiology, Chinese Academy of Sciences)

Prof. Xingyin Liu (Nanjing Medical University)

Prof. Yong‐Xin Liu (Agricultural Genomics Institute at Shenzhen, Chinese Academy of Agricultural Sciences)

Prof. Yulan Liu (Peking Universiy)

Prof. Zhanju Liu (Shanghai Tenth People's Hospital, School of Medicine, Tongji University)

Prof. Zhihua Liu (Tsinghua University)

Prof. Yongzhan Nie (Air Force Medical University)

Prof. Noah Palm (Yale School of Medicine)

Prof. An Pan (Huazhong University of Science and Technology)

Prof. Hanping Shi (Beijing Shijitan Hospital, Capital Medical University)

Prof. Jun Wang (Institute of Microbiology, Chinese Academy of Sciences)

Prof. Yan Wang (Chinese Academy of Medical Sciences/Peking Union Medical College)

Prof. Sunny Wong (Nanyang Technological University)

Prof. Kaichun Wu (Air Force Medical University)

Prof. Fengjiao Xin (Chinese Academy of Agricultural Sciences)

Prof. Jian Xu (Qingdao Institute of Bioenergy and Bioprocess Technology, Chinese Academy of Sciences)

Prof. Ruifu Yang (Beijing Institute of Microbiology and Epidemiology, Academy of Military Medical Sciences)

Prof. Yanpo Yao (Ministry of Agriculture and Rural Affairs)

Prof. Jun Yu (Chinese University of Hong Kong)

Prof. Zhengquan Yu (China Agricultural University)

Prof. Qiang Zeng (Chinese PLA General Hospital)

Prof. Chenhong Zhang (Shanghai Jiao Tong University)

Prof. Faming Zhang (Second Affiliated Hospital of Nanjing Medical University)

Prof. Heping Zhang (Inner Mongolia Agricultural University)

Prof. Fangqign Zhao (Beijing Institute of Life Sciences, Chinese Academy of Sciences)

Prof. Jiangchao Zhao (University of Arkansas)

Prof. Liping Zhao (Rutgers University)

Prof. Hao Zheng (China Agricultural University)

Prof. Jusheng Zheng (Westlake University)

Prof. Leming Zheng (Peking Universiy)

Prof. Hongwei Zhou (Zhujiang Hospital of Southern Medical University)

Prof. Shu Zhu (University of Science and Technology of China)

## CHINAGUT 2023 host organizations

Hosts:

Second Affiliated Hospital of Nanjing Medical University

National Clinical Medical Research Center for Digestive Diseases (Xi'an)

Beijing Rexinchang Biotechnology Research Institute

Gut Microbiome Academic Subgroup, Biophysical Society of China

Invited Hosts:

China National Convention Center

*iMeta*

Specialized Committee on Microbiome, Chinese Association for Microbiology

Cohosts:

Integrative Medicine Branch, Chinese Medical Doctor Association

Beijing Institutes of Life Science, Chinese Academy of Sciences

Institute of Microbiology, Chinese Academy of Sciences

Sir Run Run Hospital, Nanjing Medical University

Chinese Institute of Development Strategy for Integrative Medicine

China Medicine and Microecology Integration Alliance

Colleges of Animal Science and Technology and Animal Medicine, Huazhong Agricultural University

Specialized Committee on Germ‐Free Animals, Chinese Association for Laboratory Animal Sciences

State Key Laboratory of Digestive Disease, Chinese University of Hong Kong

Medical Center for Microecology, Southern Medical University

Microecology Branch, Guangdong Precision Medicine Application Association

Integrative Oncology Branch, China Anticancer Association

*Medicine in Microecology*

Minfu Social Welfare Foundation

State Key Laboratory of Microbial Technology, Shandong University

The first Affiliated Hospital, Sun Yat‐sen University

Tenth People's Hospital of Tongji University

Jinfeng Laboratory

*Chinese Medical Journal*

*mLife*

*hLife*

*Engineering Microbiology*

Organizer:

R Institute
